# Experiences with low-intervention clinical trials—the new category under the European Union Clinical Trials Regulation

**DOI:** 10.1177/17407745241309293

**Published:** 2025-01-22

**Authors:** Amos J. de Jong, Helga Gardarsdottir, Yared Santa-Ana-Tellez, Anthonius de Boer, Mira GP Zuidgeest

**Affiliations:** 1 Division of Pharmacoepidemiology and Clinical Pharmacology, Utrecht Institute for Pharmaceutical Sciences, Utrecht University, Utrecht, The Netherlands; 2 Division Laboratory and Pharmacy, Department of Clinical Pharmacy, University Medical Center Utrecht, Utrecht, The Netherlands; 3 Faculty of Pharmaceutical Sciences, University of Iceland, Reykjavik, Iceland; 4 Dutch Medicines Evaluation Board, Utrecht, The Netherlands; 5 Julius Center for Health Sciences and Primary Care, University Medical Center Utrecht, Utrecht, The Netherlands

**Keywords:** low-intervention clinical trials, regulatory science, European Clinical Trials regulation, pragmatic trials, investigator-initiated trials, risk-based approach

## Abstract

**Background/Aims:**

Low-intervention clinical trials have been established under the European Union Clinical Trials Regulation (EU 536/2014) which aims to simplify the conduct of clinical trials with authorized medicinal products. There is limited experience with conducting low-intervention trials. Therefore, this study aims to report on experiences and perceived (dis)advantages of low-intervention trials.

**Methods:**

We surveyed representatives of all individual clinical trials registered on the public website of the European Union Clinical Trials Information System between 31 January 2022 and 1 December 2023 that evaluated authorized investigational medicinal products and had at least one investigative site in the European Union. These representatives were approached between June 2023 and January 2024.

**Results:**

We received 70 responses (response rate 21%). Of the respondents, 31 represented a trial registered as low-intervention trial, and 39 represented a trial not registered as a low-intervention trial (hereafter “regular trials”). Simplified clinical trial monitoring and an easier regulatory approval process were perceived as the main advantages of low-intervention trials, with respectively 44% and 34% of the respondents indicating this to be an advantage in low-intervention trials. However, the respondents experienced that stringent and unclear regulatory requirements impeded the conduct of low-intervention trials. Respondents involved with regular trials indicated that 39% of the regular trials met the criteria of a low-intervention trial but were not registered as such, among others due to unfamiliarity with this trial category.

**Conclusions:**

We argue that the simplified procedures for low-intervention trials should be more detailed—for example in regulatory guidance—in the future to further simplify the conduct of clinical trials with authorized investigational medicinal products.

## Introduction

Clinical trials that evaluate authorized drugs are essential to answer questions to optimize the use of these drugs in clinical practice, such as those related to deprescribing, dose optimization, determining the line of therapy, determining the target population, drug repurposing, and cost-effectiveness. Investigator-initiated clinical trials play an important role in answering these clinical research questions that intend to optimize clinical practice. Multinational investigator-initiated trials are important to generate scientific evidence for the benefit of European Union (EU) citizens, as is also recognized by the Accelerating Clinical Trials initiative in the European Union.^1^ However, of the clinical trials that have been authorized through the EU clinical trials information system with a non-commercial sponsor, the proportion of trials that was conducted in more than one country has been stable over time and was only 9% in March 2024.^2^ In comparison, 75% of the commercial clinical trials were multinational trials in March 2024.^2^ Conducting clinical trials that evaluate authorized drugs may be impeded by administrative burden, complex (unharmonized) regulatory and safety reporting requirements, suboptimal trial processes, such as excessive trial monitoring and drug supply, and inadequate funding.^3–5^

As an example, investigators of European multinational pediatric clinical trials evaluating authorized drugs in an open-label setting have previously reported on challenges with investigator-initiated trials.^6,7^ Specifically, the investigators mentioned that they had to comply with specific drug labeling requirements, whereas these trials could have benefited from labeling in accordance with routine practice, allowing sites and local pharmacies to use their own stocks.^6,7^ To simplify the conduct of clinical trials that investigate authorized products, EU policymakers have introduced the “low-intervention clinical trials” category in the EU Clinical Trials Regulation 536/2014,^8^ which became applicable on 31 January 2022, when the clinical trials information system became operational.^9^ The Clinical Trials Regulation defines low-intervention clinical trials, as clinical trials that “pose only a minimal additional risk to subject safety compared to normal practice” and evaluate investigational medicinal products that are authorized—in accordance with the marketing authorization or evidence-based—while the diagnostic or monitoring procedures do not pose more than minimal additional risk or burden to the safety of the trial participants compared to normal clinical practice (Article 2, EU 536/2014). These trials are subject to less stringent regulatory requirements, including simplified procedures related to informed consent, clinical trial monitoring, and labeling and traceability of investigational medicinal products.^8^

Despite the expected advantages, there is currently limited experience with setting up and conducting low-intervention trials, which may impede the category’s full potential from being realized. Therefore, this study aims to report on the experiences with, and perceived (dis)advantages of, low-intervention trials.

## Methods

### Study design

We conducted a cross-sectional survey to solicit the experiences with, and perceived (dis)advantages of, low-intervention trials of clinical trial sponsors. The CROSS checklist was used to report on the study design and results (Supplemental Material 1).

### Study population

Representatives of all clinical trials that evaluated authorized investigational medicinal product with at least one site in the EU and registered in the clinical trials information system were eligible to participate in this survey. Contact information of clinical trial sponsor representatives—specifically, the primary public contacts and primary scientific contacts (hereafter referred to as “representatives” and, when applicable, “respondents”)—was retrieved for trials that investigated authorized investigational medicinal products and were submitted to the clinical trials information system between 31 January 2022 (when the clinical trials information system went live, https://euclinicaltrials.eu/) and 1 December 2023 using the R package *ctrdate* version 1.16.0.^10^

### Survey development

We designed two surveys in English; one targeting representatives involved in trials registered as low-intervention trials and another targeting representatives involved in clinical trials that were not labeled as a low-intervention trial but also investigated an authorized investigational medicinal product (hereafter, regular trials). The surveys were pretested and reviewed within the research team for validity and to evaluate the survey functionality. The survey questions were informed by the potential advantages of low-intervention trials as mentioned in the Clinical Trials Regulation. Specifically, the surveys contained open questions soliciting the experienced or expected (dis)advantages of low-intervention trials and closed questions on the simplified procedures regarding informed consent (Article 30 of EU 536/2014), clinical trial monitoring (Article 48 of EU 536/2014), investigational medicinal product handling (Article 51 of EU 536/2014), the clinical trial master file (Article 57 of EU 536/2014), and damage compensation (Article 76 of EU 536/2014) informed by the propositions on simplified procedures for low-intervention trials as described in the Clinical Trials Regulation. In addition, we added simplified assessments by national competent authorities and ethics committees as an advantage. The survey targeting the representatives of regular trials furthermore included questions to elucidate familiarity with low-intervention trials and whether the regular trial met the low-intervention trial criteria. The surveys probed the respondents to explain the experienced or perceived (dis)advantages. The surveys are available in Supplemental Materials 2 and 3.

### Data collection

Surveys were shared via email with representatives of authorized (low-intervention) clinical trials using Qualtrics software (Provo, Utah, USA) between June 2023 and January 2024. Representatives were contacted using a standardized information letter with a link to the questionnaire. A minimum of two reminders were shared via email. Representatives were also given the possibility to provide their answers orally during a structured interview, but this possibility was not used. This study concerned a descriptive study that intended to capture many perspectives and therefore, the survey was broadly distributed and no formal sample size was calculated. Trial characteristics—including trial phase, multicenter, multinational, Member States concerned, participant type, participant age range, and therapeutic area—were obtained from the public clinical trials information system website.

### Data analysis

Data were analyzed using Excel^®^. Descriptive statistics were used to report on the closed questions, using the total number of responses as the denominator unless specified otherwise. Open questions were analyzed through content analysis, by categorizing the written text according to the concepts of interest and stratifying the advantages and disadvantages.^11^

### Ethics

Written informed consent was obtained from the respondents (included in the Supplemental Materials 2 and 3). Only the research team had access to the survey data and disseminated results were aggregated and anonymized. This study did not undergo an ethics review, in accordance with applicable Dutch law, as we did not conduct a medical study.

## Results

### Respondent characteristics

Of the 117 low-intervention trials and 218 regular trial representatives who were invited, 31 (26%) and 39 (18%) responded, respectively. Of the 39 representatives of regular clinical trials, 26 (66%) indicated to be familiar with low-intervention trials and 19 (53%) had experience with submitting or conducting a low-intervention trial. Representatives of low-intervention trials reported that 22 (71%) of the submitted low-intervention trials were originally designed as low-intervention trials. All respondents represented a non-commercial trial sponsor. Table 1 presents the trial characteristics of the low-intervention trials and regular clinical trials.

**Table 1.** Characteristics of the trials the respondents were involved in.

Characteristic		Low-intervention trials (n = 31)	Regular trial (n = 39)
Trial phase	Phase 1	0 (0%)	3 (8%)
	Phase 2	1 (3%)	9 (23%)
	Phase 2/3	0 (0%)	1 (3%)
	Phase 3	7 (23%)	14 (36%)
	Phase 3/4	6 (19%)	1 (3%)
	Phase 4	17 (55%)	11 (28%)
Multicenter trial		21 (68%)	23 (59%)
Multinational trial		5 (16%)	7 (18%)
Countries involved^a,b^	Netherlands	7 (23%)	11 (28%)
	Denmark	8 (26%)	8 (21%)
	Belgium	6 (19%)	7 (18%)
	Spain	5 (16%)	4 (10%)
	France	5 (16%)	3 (8%)
	Germany	3 (10%)	5 (13%)
	Sweden	2 (6%)	7 (18%)
	Norway	1 (3%)	7 (18%)
	Italy	2 (6%)	4 (10%)
Trial participant type	Patients	28 (90%)	33 (85%)
	Healthy volunteers	1 (3%)	6 (15%)
	Both	2 (6%)	0 (0%)
Trial participant age range^a^ (years)	<18	1 (3%)	2 (5%)
	18–64	28 (90%)	37 (95%)
	≥65	28 (90%)	29 (75%)
Therapeutic area^a^	Cardiovascular diseases	7 (23%)	6 (13%)
	Neoplasms	3 (10%)	8 (21%)
	Virus diseases	4 (13%)	5 (13%)
	Respiratory tract diseases	3 (10%)	4 (10%)
	Urogenital diseases	2 (6%)	5 (13%)
	Immune system diseases	4 (13%)	0 (0%)
	Nervous system diseases	1 (3%)	5 (13%)
	Others	13 (42%)	12 (31%)

a These categories are not mutually exclusive.

b Other countries include Austria, Czechia, Finland, Greece, Hungary, Ireland, Luxembourg, Poland, Portugal, Slovakia, and the United Kingdom

### Advantages of low-intervention clinical trials

**Figure 1.**
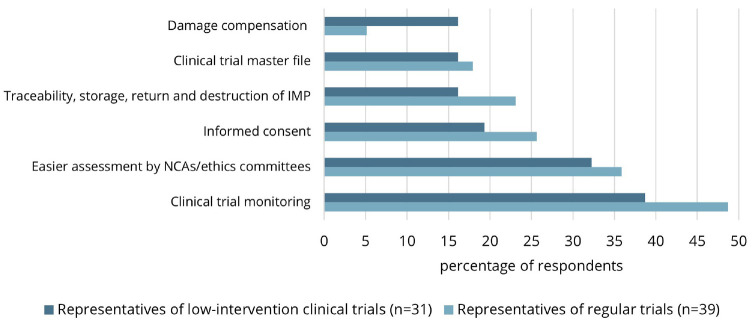
Experienced and envisioned simplified procedures of low-intervention clinical trials. IMP, investigational medicinal product; NCA, national competent authority.

Figure 1 shows the experienced or expected advantages of low-intervention trials as solicited in closed questions stratified by type of respondent. Simplified clinical trial monitoring was considered an advantage of low-intervention trials by 31 (44%) of the respondents. Although no extensive explanation was given, respondents indicated that less monitoring was necessary and that monitoring could be shifted from onsite to remote/centralized monitoring in low-intervention trials. Although not explicitly mentioned in the Clinical Trials Regulation, 24 (34%) of the respondents indicated that low-intervention trials benefit from an easier assessment by national competent authorities/ethics committees. Respondents explained that fewer documents were, and should be, required for low-intervention trials—for example, documents regarding the safety profile of the investigational medicinal product or the evaluation of pregnancies in participants.

Regarding informed consent procedures, 16 (22.9%) of the respondents experienced or expected this to be simplified in low-intervention trials. Specifically, respondents mentioned that low-intervention trials will facilitate an at-home consent procedure. One representative of a low-intervention trial experienced that consent could be obtained using an online portal and videos without actual contact with the investigative staff. Another representative indicated that, based on their experience, there is no need to obtain individual informed consent in a low-intervention cluster randomized control trial (RCT) in which hospitals are randomized. Furthermore, one respondent indicated that low-intervention trials should allow for shorter informed consent forms.

Notably, 14 (20.0%) of the respondents indicated that the traceability, storage, return, and destruction of the investigational medicinal product can be simplified in low-intervention trials. Respondents explained that these processes could take place in line with routine clinical practice (e.g. through community pharmacies) in low-intervention trials, avoiding extra work and administration. As for the clinical trial master file and damage compensation, 12 (17.1%) and 7 (10.0%) of the respondents experienced or expected these processes to be simplified in low-intervention trials. Regarding the former, the investigator brochure can be replaced by the Summary of Product Characteristics, as experienced by one respondent.

In addition to the probed advantages, several respondents indicated in open questions that low-intervention trials may reduce the administrative burden and costs due to a simpler protocol. In turn, some respondents mentioned that it was easier to recruit and involve participants and investigative sites. The respondents indicated that the low-intervention trial category can be considered in particular for pragmatic clinical trials evaluating the comparative effectiveness of an intervention in routine clinical care. In line, low-intervention trials were thought to be advantageous to study new indications of authorized medicines and to evaluate the optimal use of medication.

### Disadvantages of low-intervention clinical trials

Furthermore, experienced and expected disadvantages of low-intervention trials were solicited in open questions. Many respondents indicated that a high administrative burden due to the (relatively) new clinical trials information system and the long timelines impede the setup and conduct of low-intervention trials. In addition, stringent and unclear regulatory requirements were mentioned. As an example, one respondent indicated that only medical doctors were permitted to enroll patients in the respective member state, whereas trained nurses and medical students should also be allowed to enroll patients. Furthermore, some respondents experienced that regulators seem to focus on the risk of the intervention without looking at the risks associated with standard of care procedures. Some respondents also experienced that it is not clear which trials would fall under the definition of a low-intervention trial and what type of supporting data is needed to evaluate a well-established medicine for another indication.

The difficulty in aligning clinical trial conduct with routine care, the inability to blind in a low-intervention trial [NB: this is not detailed in the Clinical Trials Regulation], difficulty enrolling vulnerable patient groups (e.g. pediatrics), and the need for additional sponsoring when the use of the investigational medicinal product falls outside the reimbursement criteria further limited the implementation of low-intervention clinical trials, as mentioned by some the respondents.

### Regular trials that meet the low-intervention criteria

For regular clinical trials, we also solicited whether these trials met the criteria of a low-intervention trial. Of the 28 clinical trials for which this question was answered by the respondents, 11 (39%) met the low-intervention trial criteria but were not registered as such. Reasons for not registering the clinical trial as a low-intervention trial included unfamiliarity with this trial category, a limited understanding of the low-intervention trial definition (e.g. that this trial category applies to interventional research), not wanting to risk incorrectly classifying the trial as a low-intervention trial, initiation of the trial before the Clinical Trials Regulation became applicable (a transition trial), because the evidence on the efficacy was based on animal studies, and because the investigational medicinal product was used outside the conditions of the marketing authorization.

## Discussion

Clinical trial sponsors may experience difficulty implementing novel clinical trial approaches such as low-intervention trials, when limited information on what is allowed under these approaches is available. Therefore, we aimed to report on specific experiences and perceived (dis)advantages of low-intervention trials. We found that reduced clinical trial monitoring and an easier regulatory approval process are the main experienced and anticipated advantages of low-intervention trials. Although we provided a short overview of examples of simplified procedures in low-intervention trials, it was observed that specific experiences were only reported to a limited extent, highlighting the need for detailed information on the simplified procedures that can be considered for low-intervention trials. This lack of information on possible advantages may (partially) explain why a substantial proportion (39%) of the regular clinical trials conducted with authorized investigational medicinal product that met the criteria of a low-intervention trial was not registered as such.

Patrick-Brown et al.^12^ have previously argued for further specification of the low-intervention trial definition, for example, to clarify when a study should be considered a low-intervention trial or an observational study when participants are not randomized to authorized investigational medicinal product. In addition, ambiguity regarding the extent of the evidence base to consider an intervention “evidence-based” when authorized investigational medicinal products are studied outside the terms of the marketing authorization has been signaled before.^7^ Regarding the type of trials that could qualify as a low-intervention trial, regulators may emphasize the risks of the intervention as compared to the risks associated with standard of care procedures, according to the experiences of some respondents in the current study. Therefore, it should be acknowledged that standard of care and well-established interventions are not risk-free and “minimal *additional* risk compared to normal clinical practice” (Article 2, EU 536/2014) should not be interpreted as “minimal risk” by regulators when evaluating clinical trial applications.

Simplified informed consent procedures can be considered in single-country low-intervention cluster RCTs (Article 30, EU 536/2014). Previously, concerns have been expressed stemming from ambiguous interpretation of this stipulation.^13^ Namely, it has been argued that simplified consent procedures could be interpreted as an opt-out—where participants are included in the clinical trial unless they explicitly decline to participate—which may lead to inadequate ethical protection of trial participants.^13^ In the current study, one respondent indeed reported on a low-intervention trial in which no individual informed consent had to be obtained, with randomization taking place at the hospital level. In addition, simplified consent procedures could involve obtaining informed consent remotely and asynchronously, as mentioned by the respondents in the current study and should be considered by clinical trial sponsors and regulators. Moreover, simplified consent procedures, including waivers, could be considered for non-cluster trials as has previously been argued by Dal-Ré et al.^14,15^

More information on the simplified procedures that can be considered for low-intervention trials could be communicated by sponsors, sharing their experience through scientific and gray literature. In addition, regulatory guidance could furthermore detail (1) how low-intervention trials can be considered in vulnerable groups—including participants who are incapable of giving consent personally (e.g. EUCT: 2022-500717-64-00) and pregnant women (e.g. EUCT: 2022-500933-10-00; 2022-501142-30-00), (2) how damage compensation procedures can differ from regular clinical trials, and (3) whether and to what extent trial-specific training is necessary for health care professionals beyond the principal investigator involved in the trial conduct. Regarding the latter, health care professionals, including general practitioners and community pharmacists, involved with low-risk trial-related tasks should not always be required to have Good Clinical Practice training, particularly in the context of low-intervention trials.^7^

### Strengths and limitations

To our knowledge, this is the first study that reports on the experiences with, and perceived (dis)advantages of the new category of “low-intervention clinical trials” as detailed in the Clinical Trials Regulation (EU 536/2014). Through a survey that was broadly distributed to all sponsor representatives that conducted a trial with authorized investigational medicinal product, we were able to quantify and describe the experienced and perceived (dis)advantages of low-intervention trials. The results of this study illustrate the need for more clarity regarding the added benefit of this trial category and can thereby inform future policy and regulatory guidance to facilitate the conduct of clinical trials with authorized medicinal products.

The current study is subject to several limitations. First, since the survey was distributed only in English, potential participants who were not fluent in English may have chosen not to participate, potentially limiting the geographical diversity. In addition, not all respondents provided in-depth feedback on experienced and anticipated (dis)advantages. This may be due to participants’ limited experience with conducting low-intervention trials or may be reflective of the uncertain benefit of this trial category. Since the conduct of the study, more clinical trials with authorized investigational medicinal product, including low-intervention trials, have been approved in the clinical trials information system and future studies could explore the trends in uptake over time and evaluate future experiences, when more insight is gained into the benefit of using this trial category.

## Conclusion

In the current study, we describe some examples of simplified procedures that can be considered for low-intervention trials. Increased awareness of, and guidance on, the possibilities of simplified procedures in a low-intervention trial will facilitate the conduct of (multinational) clinical trials that aim to improve clinical practice by evaluating authorized investigational medicinal products.

## Supplemental Material

sj-docx-1-ctj-10.1177_17407745241309293 – Supplemental material for Experiences with low-intervention clinical trials—the new category under the European Union Clinical Trials Regulation Supplemental material, sj-docx-1-ctj-10.1177_17407745241309293 for Experiences with low-intervention clinical trials—the new category under the European Union Clinical Trials Regulation by Amos J. de Jong, Helga Gardarsdottir, Yared Santa-Ana-Tellez, Anthonius de Boer and Mira GP Zuidgeest in Clinical Trials

sj-docx-2-ctj-10.1177_17407745241309293 – Supplemental material for Experiences with low-intervention clinical trials—the new category under the European Union Clinical Trials Regulation Supplemental material, sj-docx-2-ctj-10.1177_17407745241309293 for Experiences with low-intervention clinical trials—the new category under the European Union Clinical Trials Regulation by Amos J. de Jong, Helga Gardarsdottir, Yared Santa-Ana-Tellez, Anthonius de Boer and Mira GP Zuidgeest in Clinical Trials

sj-docx-3-ctj-10.1177_17407745241309293 – Supplemental material for Experiences with low-intervention clinical trials—the new category under the European Union Clinical Trials Regulation Supplemental material, sj-docx-3-ctj-10.1177_17407745241309293 for Experiences with low-intervention clinical trials—the new category under the European Union Clinical Trials Regulation by Amos J. de Jong, Helga Gardarsdottir, Yared Santa-Ana-Tellez, Anthonius de Boer and Mira GP Zuidgeest in Clinical Trials
