# Supplementary material for: Experiences with low-intervention clinical trials—the new category under the European Union Clinical Trials Regulation
Source: Clin Trials. 2025 Jan 22;22(4):494–500. doi: 10.1177/17407745241309293 (PMC12318156; doi:10.1177/17407745241309293)
Supplement: sj-docx-3-ctj-10.1177_17407745241309293 – Supplemental material for Experiences with low-intervention clinical trials—the new category under the European Union Clinical Trials Regulation [file sj-docx-3-ctj-10.1177_17407745241309293.docx]

Survey – non-low-intervention clinical trials

Dear ${e://Field/RecipientFirstName} ${e://Field/RecipientLastName},
  
You have been invited to take part in a survey into low-intervention clinical trials. It is important for the participant to understand the purpose of this survey and what it entails. Please carefully read the information below.

**Purpose of the research**
Through this survey we aim to solicit the perspective of clinical trial sponsors and investigators on ‘low-intervention clinical trials’. We are also particularly interested in the perspective of sponsors that have limited or no experience with conducting low-intervention clinical trials.

**Procedure and data protection**
The data obtained from this survey will be stored and kept within the digital environment of the Utrecht University. Only the research group will have access to the raw data. The researchers aim to disseminate the results of this survey through a peer-reviewed scientific publication. Disseminated results or quotes will never contain personal information that could reasonably identify respondents.

**Voluntary participation**
Your participation is highly appreciated. Participating in this survey is voluntarily and you are free to withdraw at all times, also after consenting to participate. You are free to withdraw without consequences or providing a reason. Withdrawal of informed consent may not affect analyzed data collected prior to the withdrawal of consent.

If you have any questions, please contact us via this e-mail address: [email address]

I understand the information presented in this form. I understand that my participation is voluntary.

- I do consent (1)

Name

________________________________________________________________

Q Do you prefer to provide input by teleconference or phone?

- No, continue the survey (1)
- Yes (4)

Display This Question:

If Do you prefer to provide input by teleconference or phone? = Yes

Q Please provide a telephone number or e-mail address that we can reach you on.

________________________________________________________________

Thank you for participating in our study. We will contact you as soon as possible.

1. For which organization do you currently work?

________________________________________________________________

2. We are contacting you in relation to ${e://Field/ExternalDataReference}. Is it correct that you are - or have been - involved in this trial?

- Yes (1)
- No (2)

Display This Question:

If We are contacting you in relation to ${e://Field/ExternalDataReference}. Is it correct that you a... = No

Q Could you provide us with contact information of a representative who is/was involved in the conduct of ${e://Field/ExternalDataReference}?

________________________________________________________________

Q Are you familiar with ‘low-interventional clinical trials’?

- Yes (1)
- No (2)

Q Do you have experience with submitting a low-intervention trial in the Clinical Trials Information System or do you have experience with conducting a low-intervention clinical trial? more information

- Yes (1)
- No (2)

Q In your opinion, what are the (possible) difficulties or disadvantages of setting-up and conducting low-intervention clinical trials? And could you explain why?

________________________________________________________________

________________________________________________________________

________________________________________________________________

________________________________________________________________

________________________________________________________________

Q In your opinion, what are the (possible) advantages of conducting low-intervention clinical trials? And could you explain why?

________________________________________________________________

________________________________________________________________

________________________________________________________________

________________________________________________________________

________________________________________________________________

Q Which of the following advantages regarding the setup and conduct of a low-intervention clinical trial would you anticipate because of its “low-intervention” character? Please select all that apply.

- Simplified means of informed consent for cluster randomized trials more information function myFunction1() {alert("Informed consent may be obtained by simplified means if, amongst others, the cluster randomized trial is a low-intervention clinical trial and the investigational medicinal products are used in accordance with the terms of the marketing authorization (article 30 Regulation (EU) No 536/2014).");} (1)

Display This Question:

If Which of the following advantages regarding the setup and conduct of a low-intervention clinical... = Simplified means of informed consent for cluster randomized trials more information function myFunction1() {alert("Informed consent may be obtained by simplified means if, amongst others, the cluster randomized trial is a low-intervention clinical trial and the investigational medicinal products are used in accordance with the terms of the marketing authorization (article 30 Regulation (EU) No 536/2014).");}

Q Could you elaborate and explain why you anticipate this advantage?

________________________________________________________________

________________________________________________________________

________________________________________________________________

________________________________________________________________

________________________________________________________________

Q

- Monitoring of the trial conduct more information function myFunction2() { alert("In order to verify that the rights, safety and well-being of subjects are protected, that the reported data are reliable and robust, and that the conduct of the clinical trial is in compliance with the requirements of this Regulation, the sponsor shall adequately monitor the conduct of a clinical trial. The extent and nature of the monitoring shall be determined by the sponsor on the basis of an assessment that takes into consideration all characteristics of the clinical trial, including whether the clinical trial is a low-intervention clinical trial (article 48 Regulation (EU) No 536/2014)."); } (1)

Display This Question:

If   = Monitoring of the trial conduct more information function myFunction2() { alert("In order to verify that the rights, safety and well-being of subjects are protected, that the reported data are reliable and robust, and that the conduct of the clinical trial is in compliance with the requirements of this Regulation, the sponsor shall adequately monitor the conduct of a clinical trial. The extent and nature of the monitoring shall be determined by the sponsor on the basis of an assessment that takes into consideration all characteristics of the clinical trial, including whether the clinical trial is a low-intervention clinical trial (article 48 Regulation (EU) No 536/2014)."); }

Q Could you elaborate and explain why you anticipate this advantage?

________________________________________________________________

________________________________________________________________

________________________________________________________________

________________________________________________________________

________________________________________________________________

Q

- Traceability, storage, return and destruction of investigational medical product more information function myFunction3() { alert("Investigational medicinal products shall be stored, returned and/or destroyed as appropriate and proportionate to ensure the safety of the subject and the reliability and robustness of the data generated in the clinical trial, in particular, taking into account whether the investigational medicinal product is an authorized investigational medicinal product, and whether the clinical trial is a low-intervention clinical trial (article 51 Regulation (EU) No 536/2014)."); } (1)

Display This Question:

If   = Traceability, storage, return and destruction of investigational medical product more information function myFunction3() { alert("Investigational medicinal products shall be stored, returned and/or destroyed as appropriate and proportionate to ensure the safety of the subject and the reliability and robustness of the data generated in the clinical trial, in particular, taking into account whether the investigational medicinal product is an authorized investigational medicinal product, and whether the clinical trial is a low-intervention clinical trial (article 51 Regulation (EU) No 536/2014)."); }

Q Could you elaborate and explain why you anticipate this advantage?

________________________________________________________________

________________________________________________________________

________________________________________________________________

________________________________________________________________

________________________________________________________________

Q

- Clinical trial master file more information function myFunction4() { alert("The clinical trial master file shall at all times contain the essential documents relating to that clinical trial which allow verification of the conduct of a clinical trial and the quality of the data generated, taking into account all characteristics of the clinical trial, including in particular whether the clinical trial is a low-intervention clinical trial (article 57 Regulation (EU) No 536/2014)."); } (1)

Display This Question:

If   = Clinical trial master file more information function myFunction4() { alert("The clinical trial master file shall at all times contain the essential documents relating to that clinical trial which allow verification of the conduct of a clinical trial and the quality of the data generated, taking into account all characteristics of the clinical trial, including in particular whether the clinical trial is a low-intervention clinical trial (article 57 Regulation (EU) No 536/2014)."); }

Q Could you elaborate and explain why you anticipate this advantage?

________________________________________________________________

________________________________________________________________

________________________________________________________________

________________________________________________________________

________________________________________________________________

Q

- Damage compensation more information function myFunction5() { alert("No additional insurance, a guarantee, or a similar arrangement is required from the sponsor for low-intervention clinical trials, if any possible damage that could be suffered by a subject resulting from the use of the investigational medicinal product in accordance with the protocol of that specific clinical trial on the territory of that Member State is covered by the applicable compensation system already in place (article 76 Regulation (EU) No 536/2014)."); } (1)

Display This Question:

If   = Damage compensation more information function myFunction5() { alert("No additional insurance, a guarantee, or a similar arrangement is required from the sponsor for low-intervention clinical trials, if any possible damage that could be suffered by a subject resulting from the use of the investigational medicinal product in accordance with the protocol of that specific clinical trial on the territory of that Member State is covered by the applicable compensation system already in place (article 76 Regulation (EU) No 536/2014)."); }

Q Could you elaborate and explain why you anticipate this advantage?

________________________________________________________________

________________________________________________________________

________________________________________________________________

________________________________________________________________

________________________________________________________________

Q

- Easier assessment by national competent authorities/ethics committees envisioned when trial is labelled as low-intervention (1)

Display This Question:

If   = Easier assessment by national competent authorities/ethics committees envisioned when trial is labelled as low-intervention

Q Could you elaborate and explain why you anticipate this advantage?

________________________________________________________________

________________________________________________________________

________________________________________________________________

________________________________________________________________

________________________________________________________________

Q

- None of the above (1)

Q Which of the following is true for ${e://Field/ExternalDataReference}? Please select all that apply

- The investigational medicinal products, excluding placebos, used in this trial are authorized
- The investigational medicinal products are used according to marketing authorization
- The use of the investigational medicinal products is evidence based
- The additional diagnostic or monitoring procedures do not pose more than minimal additional risk or burden to the safety of the subjects compared to normal clinical practice in any member state concerned
- None of the above

Display This Question:

If Which of the following is true for ${e://Field/ExternalDataReference}? Please select all that apply = The use of the investigational medicinal products is evidence based

Q How could you show to the authorities that the use of the IMP(s) is evidence based?

________________________________________________________________

________________________________________________________________

________________________________________________________________

________________________________________________________________

________________________________________________________________

Display This Question:

If Which of the following is true for ${e://Field/ExternalDataReference}? Please select all that apply = The additional diagnostic or monitoring procedures do not pose more than minimal additional risk or burden to the safety of the subjects compared to normal clinical practice in any member state concerned

Q How could you show to the authorities that the additional diagnostic or monitoring procedures do not pose more than minimal additional risk or burden?

________________________________________________________________

________________________________________________________________

________________________________________________________________

________________________________________________________________

________________________________________________________________

Display This Question:

If Which of the following is true for ${e://Field/ExternalDataReference}? Please select all that apply = The investigational medicinal products, excluding placebos, used in this trial are authorized

And Which of the following is true for ${e://Field/ExternalDataReference}? Please select all that apply = The investigational medicinal products are used according to marketing authorization

And Which of the following is true for ${e://Field/ExternalDataReference}? Please select all that apply = The additional diagnostic or monitoring procedures do not pose more than minimal additional risk or burden to the safety of the subjects compared to normal clinical practice in any member state concerned

Or Which of the following is true for ${e://Field/ExternalDataReference}? Please select all that apply = The investigational medicinal products, excluding placebos, used in this trial are authorized

And Which of the following is true for ${e://Field/ExternalDataReference}? Please select all that apply = The use of the investigational medicinal products is evidence based

And Which of the following is true for ${e://Field/ExternalDataReference}? Please select all that apply = The additional diagnostic or monitoring procedures do not pose more than minimal additional risk or burden to the safety of the subjects compared to normal clinical practice in any member state concerned

Q It seems that your trial meets all the requirements for a low-intervention clinical trial. Was there a reason it was not submitted as such?

________________________________________________________________

________________________________________________________________

________________________________________________________________

________________________________________________________________

________________________________________________________________

Q If you have any additional comments or if you would like to provide more information via a follow-up call, please feel free to indicate that here.

________________________________________________________________

________________________________________________________________

________________________________________________________________

________________________________________________________________

________________________________________________________________

Display This Question:

If If If you have any additional comments or if you would like to provide more information via a follow-up call, please feel free to indicate that here. Text Response Is Not Empty

Q If you would like to provide more information, please leave your email address or telephone number here.

________________________________________________________________

Q Please be informed that this is the final question. If you click next, the survey answers will be submitted.
